# A Dutch Survey on Medication Adjustments after Metabolic and Bariatric Surgery: Experiences of Bariatric Surgeons, Internists, Pharmacists, and General Practitioners

**DOI:** 10.1007/s11695-024-07197-2

**Published:** 2024-04-02

**Authors:** Cedric Lau, Charlotte van Kesteren, Robert M. Smeenk

**Affiliations:** 1 Department of Clinical Pharmacy, Albert Schweitzer Hospital, Dordrecht, The Netherlands; 2 Department of Surgery, Albert Schweitzer Hospital, Dordrecht, The Netherlands; 3 Department of Pharmacy and Pharmacology, Antoni Van Leeuwenhoek Hospital/The Netherlands Cancer Institute, Amsterdam, The Netherlands; 4 Department of Clinical Pharmacy, University Medical Center Utrecht, Utrecht University, Utrecht, The Netherlands

**Keywords:** Bariatric surgery, Clinical practice, Medication, Pharmacovigilance

## Abstract

**Background:**

As metabolic and bariatric surgery (MBS) can alter the pharmacokinetics of drugs, post-bariatric surgery patients may require medication adjustments and monitoring. To improve pharmacotherapy in these patients, we aimed to understand the beliefs, attitudes, knowledge, and concerns of healthcare professionals who treat these patients.

**Methods:**

A survey by means of an online questionnaire was divided into six sections. It was sent to bariatric surgeons, internists, pharmacists, and general practitioners in the Netherlands.

**Results:**

Out of 229 returned surveys, 222 were included. Virtually all respondents (98%) expected MBS to influence the effect of medication. Both reduced efficacy (23%) and more adverse events or medication-related complications (21%) were recognized. Two-thirds of the respondents felt competent to prescribe or to provide advice regarding medication in post-bariatric surgery patients.

Most of the respondents (95%) believed that other healthcare professionals should be aware of the contraindication “bariatric surgery”. Of the respondents, 37% indicated that they were not aware of the medication advice incorporated in the electronic health record systems. Almost half of the respondents (48%) indicated that they documented changes in drug effects. Most respondents answered that these ought to be registered in the pharmacovigilance database or national registry.

**Conclusions:**

The majority of prescribers and pharmacists believe that patients will receive better pharmacotherapy if healthcare professionals take MBS into account. However, not all prescribers think they are competent to act adequately. To improve this, information on changed drug effects after MBS should be more widely shared among healthcare professionals via resources that are easily accessible.

**Graphical Abstract:**

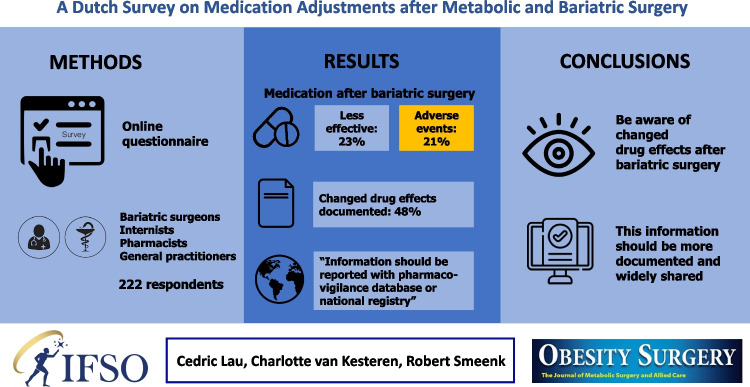

**Supplementary Information:**

The online version contains supplementary material available at 10.1007/s11695-024-07197-2.

## Introduction

Metabolic and bariatric surgery (MBS) is the most effective treatment for patients with severe obesity to lose weight and improve their quality of life. The procedure may have a significant effect on orally administered drugs that these patients use after the surgery. Information about the pharmacokinetic profile of drugs after MBS has become increasingly available [1–4].

A recent analysis reported on the worldwide documented pharmacovigilance signals associated with MBS. In this report, pharmacovigilance signals involving 165 drug reports and 128 patients were described [5]. Most signals were about Roux-en-Y gastric bypass patients (77%) and described drug ineffectiveness (75%). This low number of 165 drug reports up to 2020 globally may reflect either a low clinical impact of MBS or an underreporting of modified effects [5]. In other words, healthcare professionals may not report adverse events, complications, or reduced drug efficacy attributed to MBS. However, it is essential that information regarding changed efficacy or toxicity in patients with MBS is documented and shared among healthcare providers.

The pharmacotherapy adjustments for patients with MBS are suggested to be overlooked in clinical practice [6]. Previously, we reported on the registration of the contraindication “bariatric surgery” in a single Dutch teaching hospital to optimize pharmacotherapy [6]. The contraindication was registered in 70% of the patients in the hospital electronic health record, while this was only 28% in general practitioner (GP) practices and 25% in community pharmacies. Correct registration of this contraindication is required to automatically enable the pop-up of medication dosing advice in case of a patient with MBS. While a positive experience with the implemented drug dosing advice could increase the chance of correct registration of the contraindication, having a negative experience with drug dosing advice could demotivate healthcare providers to do so.

Overall, if healthcare professionals do not take pharmacokinetic changes after MBS into consideration, post-bariatric surgery patients may not receive optimal pharmacotherapy. To improve this, it is important to understand the beliefs, concerns, experiences, and attitudes of healthcare professionals who treat patients with MBS. To evaluate this, we aimed to explore the knowledge, experience, and beliefs of Dutch healthcare professionals by means of an electronic survey.

## Methods

### Study Design

A nationwide web-based cross-sectional survey was performed using Castor EDC (Electronic Data Capture) [7]. Healthcare professionals who are working in the fields of general medicine, internal medicine, bariatric surgery, and pharmacy (community, hospital, and outpatient pharmacy) were invited to participate by a link sent via their professional societies. Participants had the opportunity to complete the survey in the period from 15 April 2022 until 31 December 2022. Participation in this survey was completely voluntary, and the results were entered and processed anonymously.

### Questionnaire

A questionnaire was constructed that contained a brief introduction explaining the topics that would be surveyed. The questionnaire consisted of 29 questions and four sub-questions, divided into six sections. The first section concerned gathering demographic and background information of the participants. The second part (Q6–8) surveyed the responders’ beliefs about pharmacotherapy after MBS. In the third section (Q9–11), participants were asked about concerns about medication after MBS. The next section (Q12–14) consisted of questions regarding the exchange of information about MBS. In the penultimate section (Q15–25), participants were asked about pharmacotherapy monitoring after MBS. Finally, questions (Q26–29) were asked about education related to MBS and medication (Supplementary Materials 1). Participants could not enter the next section unless all questions of the previous section were answered.

### Survey Analysis

The survey responses of the participants were automatically tabulated and stored within Castor EDC [7]. The data were analyzed with the software package for statistical computing and graphics R version 4.0.5 (R Foundation for Statistical Computing, Vienna, Austria). Surveys were included when at least one question from the second section was answered. Age was coded into a 6-level covariate (< 30, 30–39, 40–49, 50–59, ≥ 60 years or not provided). The practice setting was coded into bariatric surgery, internal medicine, GP practices, and pharmacies. Pharmacists were subdivided into their working fields: community pharmacy, hospital pharmacy, or outpatient pharmacy.

Questions with ordinal answer categories were plotted using Likert plots. Most of the items were graded on a 4-point Likert scale. Open questions were first categorized into answer categories before analyzing.

## Results

### Characteristics of Respondents

The survey link was sent to approximately 5000 potential participants. In total, 229 questionnaires were returned. Of these questionnaires, 185 (80.8%) were completely filled out. Of all returned questionnaires, 222 (96.9%) were included in the analysis because they contained at least one answered question regarding the respondents’ beliefs. The survey response rate was approximately 4.4%. See Table 1 for the characteristics of the respondents. Of the responders, 67.6% were female with a median age between 30 and 39 years old. Although the questionnaire was filled out by more pharmacists than prescribers (61.7% vs 36.5%), the prescribers who responded to this questionnaire were generally more involved in daily practice with patients with MBS than pharmacists. Of the 44 bariatric surgeons and residents, 35 (80%) declared to be daily involved with the clinical care for this group.

**Table 1:** Characteristics of the respondents

	Pharmacists (*N* = 138)	Prescribers (*N* = 81)	Others (*N* = 3)
Sex, *n* (%)			
- Female (%)	105 (77)	43 (53)	2 (67)
- Male (%)	31 (22)	37 (46)	1 (33)
- Prefer not to provide	2 (1.4)	1 (1.2)	-
Age, *n* (%)			
- 20–29	26 (19)	7 (8.6)	-
- 30–39	52 (38)	27 (33)	2 (67)
- 40–49	30 (22)	24 (30)	-
- 50–59	22 (16)	16 (20)	1 (33)
- ≥ 60	7 (5.1)	5 (6.2)	-
- Prefer not to provide	1 (0.7)	2 (2.5)	-
Position, *n* (%)			
- Specialist	110 (80)	56 69)	N/A
- Resident	18 (13)	22 (27)	
- Student	2 (1.4)	-	
- Others	Pharmacist 3 (2.2) Technician 5 (3.6)	Physician Assistant or Nursing Specialist: 3 (3.7)	
Practice setting			
- Hospital	Clinical: 64 (46)	Surgery: 44 (54)	3 (100)
	Outpatient: 19 (14)	Internal medicine: 23 (28)	
- Community	51 (37)	GP: 13 (16)	-
- Not involved in patient care	4 (2.9)	1 (1.2)	-
Frequency of providing care to patients with MBS			
- Rarely	22 (16)	6 (7.4)	-
- Sometimes	70 (51)	17 21)	-
- Often	33 (24)	19 (24)	-
- Daily or almost daily	13 (9.5)	39 (48)	3 (100)

*GP* general practitioner, *MBS* metabolic and bariatric surgery

Three responders did not belong to the original intended group of pharmacists or prescribers. All of them were clinical dieticians. Since this number was low (< 5%), they were not excluded in advance. The detailed answers of the included respondents are provided in Supplementary Materials 2.

### Expectations of Pharmacotherapy in Patients with MBS

**Fig. 1:**
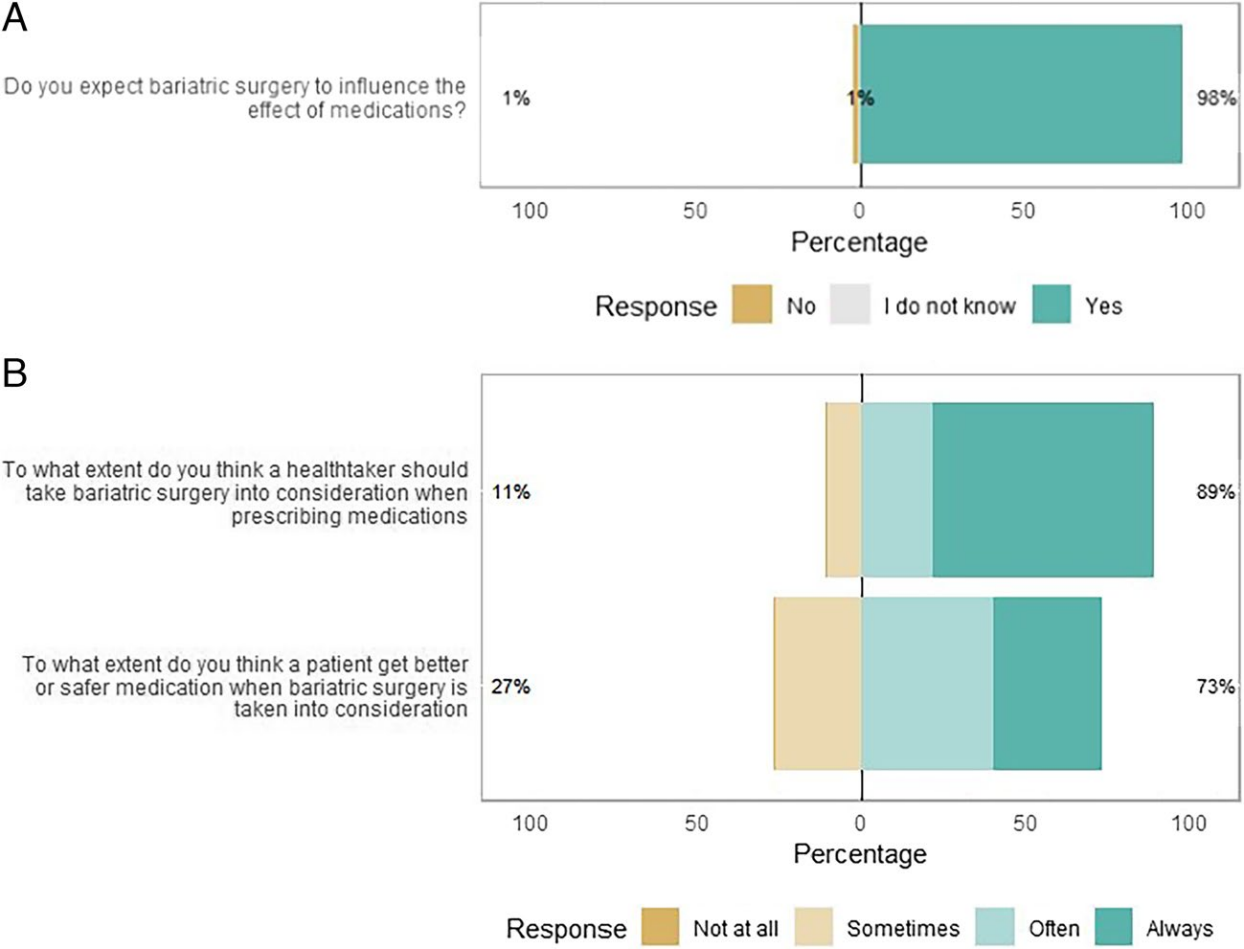
Likert plot of the healthcare providers’ expectations towards MBS. **a** Expectations of MBS to influence the effect on medication (*N* = 222). **b** Consideration of MBS by healthcare professional to improve pharmacotherapy (*N* = 222). The size of the bar is proportional to the number of respondents

Of the 222 respondents, almost all expected MBS to influence the effect of medications (Fig. 1a). There was less consensus on whether a healthcare professional should consider prior bariatric surgery status during prescribing or whether this will lead to safer pharmacotherapy for a patient after MBS (Fig. 1b).

### Concerns Regarding Medication after MBS

Of the healthcare providers, 29% often or always worried about the medication after MBS. In this survey, most healthcare providers sometimes worried about this issue and about the fact that no suitable medication would be available for patients with MBS (Supplementary Materials 3, Figure S1).

### Exchange of Information after MBS

In the Netherlands, clinical decision support for patients with MBS can be enabled once the contraindication “bariatric surgery” is registered. This contraindication was registered at least once by 63% of the respondents in the electronic health records system last year (Supplementary Materials 3, Figure S2). The registration was most frequently performed by pharmacists (99/129, 77%), followed by GPs (9/13, 69%) and bariatric surgeons (23/43, 53%). None of the internists registered the contraindication.

**Fig. 2:**
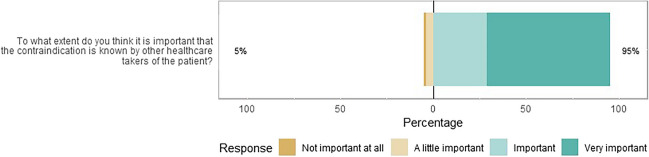
Likert plot about the beliefs towards the exchange of information related to MBS. (*N* = 207). The size of the bar is proportional to the number of respondents

Virtually all respondents thought it is important or very important that other healthcare providers of post-bariatric surgery patients be aware of the contraindication “bariatric surgery” (Fig. 2). Privacy concerns about sharing information about the contraindication bariatric surgery were indicated to be not or less important by the majority of the respondents (Supplementary Materials 3, Figure S3).

### Monitoring of Pharmacotherapy after MBS/Pharmacovigilance

**Fig. 3:**
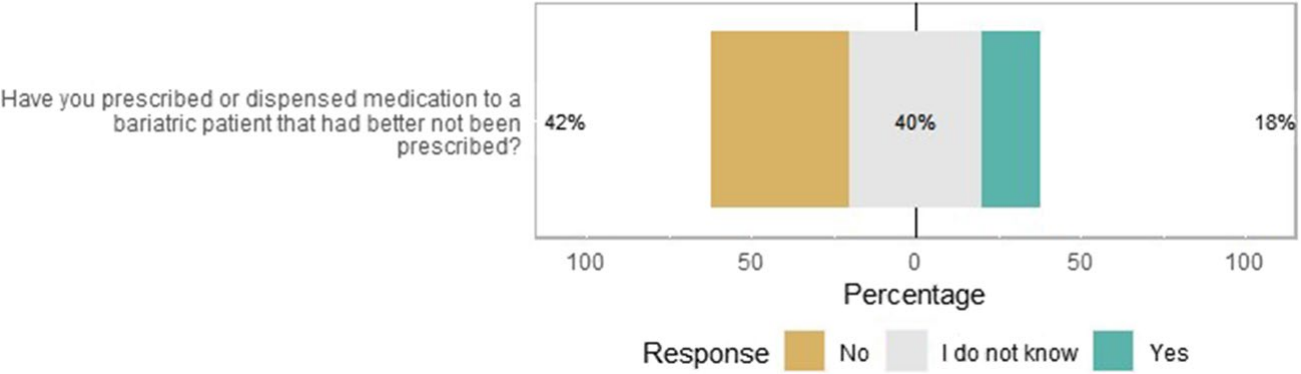
Likert plot about prescribing or dispensing drugs that had better be not prescribed to a patient with MBS (*N* = 204). The size of the bar is proportional to the number of respondents

Of the healthcare providers, 18% have prescribed or dispensed a drug to a post-bariatric surgery patient that had better be not prescribed (Fig. 3). Of the respondents, 139 healthcare providers (63%) indicated that they were aware of the medication advice incorporated in the electronic health record systems. By contrast, 15 healthcare professionals (11%) indicated not to have received any medication advice yet or that they found them unhelpful. Those who indicated that the medication advice was helpful, mentioned that it helped them to increase the efficacy of the drug (89/202, 44%), reduce adverse events (75/202, 37%), and provide drug counseling to the patient (67/202, 33%).

**Fig. 4:**
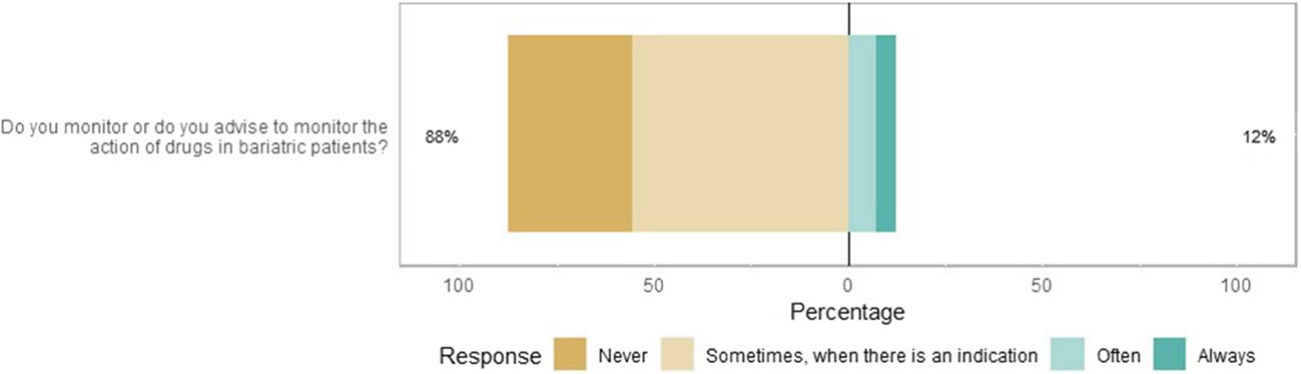
Likert plot of the monitoring of patients with MBS (*N* = 197). The size of the bar is proportional to the number of respondents

In this survey, almost 90% of the healthcare professionals answered that they never or only sometimes monitored or advised to monitor the action of drugs in patients with MBS (Fig. 4). Of the respondents who did monitor or advised to perform monitoring, most of them indicated to do so by asking about the efficacy of drugs (103/146, 71%), checking laboratory values including drug levels (98/146, 67%), and questioning about the adverse events of drugs (67/146, 46%).

Almost a quarter of the healthcare professionals (45/198, 23%) pointed out that their patients experienced less effect of their medication after MBS. None of the GPs and only several pharmacists mentioned this, while half of the internists and 28% (12/39) of the surgeons recognized this problem. Apart from vitamin depletions, the most frequently mentioned drugs belong to the class of psychotropic drugs (15 times), thyroid drugs (7 times), and oral contraceptives (3 times). One respondent mentioned antiparkinsonian drugs. In Supplementary Materials 2, an overview of all provided examples is listed.

On the other hand, 21% (40/193) of the respondents indicated that they had heard of adverse events or medication-related complications to occur in patients with MBS. This was regardless of the background of the respondent (20% of pharmacists and 25% of prescribers). The most frequently mentioned events were related to gastrointestinal disorders (5 times) and dysregulation of psychotropic drugs (2 times).

Regarding interventions in patients with post-bariatric surgery, 31/193 (16.1%) respondents had not performed any intervention or were never advised to do so. Of the performed interventions, the most frequently mentioned ones were switching to another drug (115/162, 71%), switching to another formulation (72/162, 44%), providing extra counseling (67/162, 41%), and performing additional laboratory control including drug levels (59/162, 36%). Of the healthcare professionals, 29% indicated that they often or always provided specific drug counseling after MBS (Supplementary Materials 3, Figure S4).

The changes that patients experienced were documented by 48% of the healthcare providers (Supplementary Materials 3, Figure S5). The majority of them answered that it was documented in the electronic file of the patient (87/91, 96%), followed by an electronic letter to another healthcare provider (20/91, 22%). The respondents were also asked where the information regarding efficacy, adverse events, and complications as a consequence of MBS should be documented for an individual patient. According to 36% (68/190) of the respondents, it should only be documented in the patients’ electronic health records. The majority indicated that it should be reported elsewhere as well: with The Netherlands Pharmacovigilance Centre (98/190, 52%), followed by the Dutch bariatric surgery registry (72/190, 38%). In addition, some responders indicated that such information should be accessible in handbooks (23/190, 12%) that contain information on drug dosages, indications, contraindications, and drug-drug interactions.

### Education

**Fig. 5:**
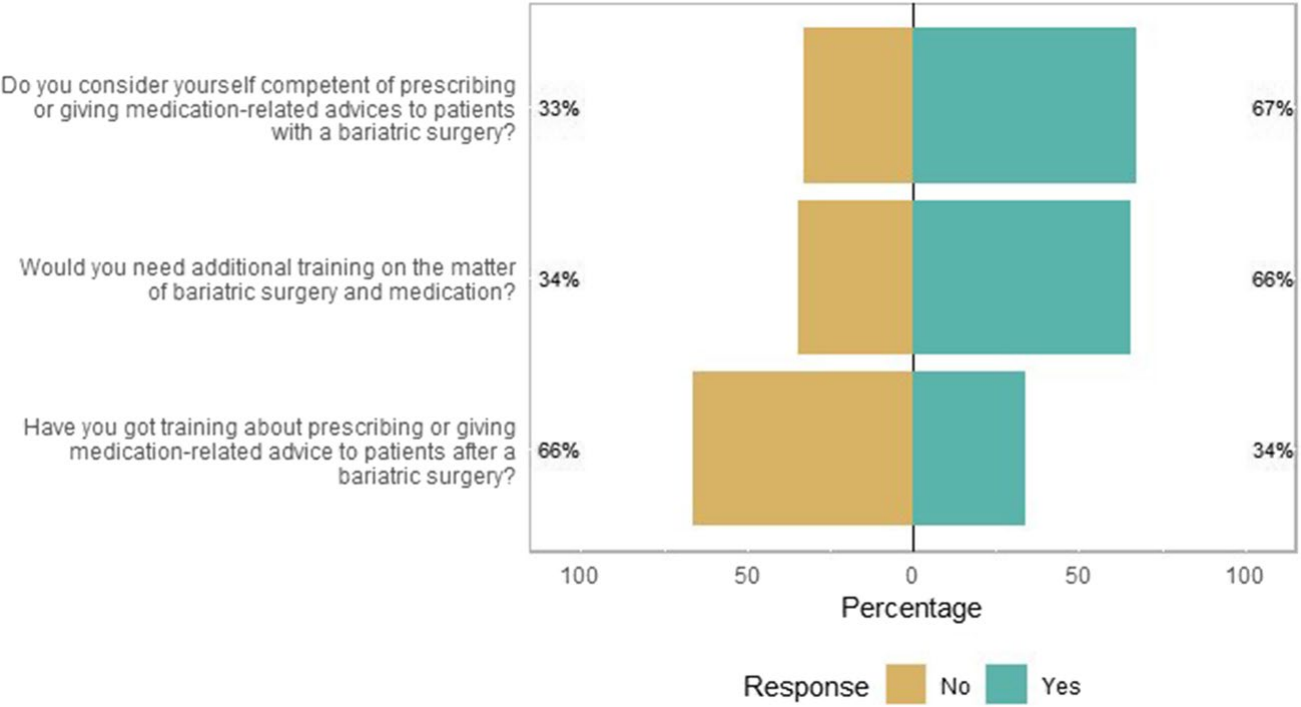
Likert plot of statements regarding training (*N* = 188). The size of the bar is proportional to the number of respondents

Two-thirds of the respondents considered themselves competent in prescribing or giving medication-related advice to patients who underwent MBS (Fig. 5). Of the surveyed participants, one-third indicated that they had followed training on prescribing drugs for or providing medication-related advice to patients with MBS. Especially pharmacists (50/115, 43%) and surgeons (11/38, 29%) indicated having followed training, while internists and GPs answered they almost had not. Two-thirds would like to have additional training on this topic. This was similar for pharmacists (73/115, 63%) and prescribers (51/73, 70%).

## Discussion

This qualitative study aimed to examine the experiences, knowledge, beliefs, and concerns of healthcare providers towards medication adjustments in post-bariatric surgery patients. It shows that most Dutch healthcare professionals believe it is important that they take MBS into account when prescribing or dispensing medication. Part of the responders thinks that this will improve the pharmacotherapy. To take it even further, some responders do worry that MBS influences pharmacokinetics/pharmacodynamics in such a way that no drugs will be available afterward.

Previously, a Brazilian survey was sent out to non-bariatric prescribers regarding drug-absorption issues [8]. While they reported that 50% of the non-bariatric clinicians believe that RYGB may interfere with drug absorption, virtually all healthcare professionals in our study believe MBS to influence the effect of drugs. The results may be different, as pharmacists were not included in the study by da Guedes et al*.* [8]. In line with our results for the internists and surgeons, da Guedes et al*.* [8] described that a substantial part of the clinicians observed some drug inefficacy in their MBS patient population. Interestingly, no GP and only a minority of the pharmacists in our survey recognized problems of reduced drug effectiveness in patients with MBS. Pharmacists in our survey tended to be less involved in care for post-bariatric surgery patients and might be less able to recognize reduced effectiveness of drugs in these patients. The question arises whether drug inefficacy does not occur in a GP setting or whether possible cases have not been recognized as being related to MBS.

Drug-related problems may occur frequently in post-bariatric surgery patients, as suggested by Wang et al*.* [9] in a retrospective study in the perioperative bariatric setting. Therefore, it is an interesting finding in the current study that post-bariatric surgery changes in the effect of drugs are reported to be almost solely documented in the patient’s electronic health record. This information on changed effects of drugs in specific patients can therefore not always be automatically shared with other healthcare professionals. Paradoxically, this highly contrasts with the belief that most surveyed healthcare professionals think it is important that other healthcare professionals be aware of their patients having undergone a bariatric surgical procedure.

To share the information of a patient having undergone a bariatric surgical procedure, it would be helpful to register the contraindication “bariatric surgery.” In line with the results of our previous report in a single center [6], more than one-third of the respondents (37%) indicated in this national survey that they have not registered the contraindication yet. A reason for this could be that they were unaware of medication advice that has already been implemented in the electronic prescribing or pharmacy information system. Therefore, it may be helpful to make prescribers aware of the accessibility to medication advice for post-bariatric surgery patients. Others indicated that the advice is not specific and does not help at all. This advocates for more specific dosing advice for high-risk drugs and the most commonly used drugs in the future for post-bariatric surgery patients.

Apart from the known changed drug effects, the participants reported interesting answers not previously reported in the literature. One of them noted a reduced effect of antiparkinsonian medication. However, the respondent did not specify which drugs were involved in this observation. Furthermore, one respondent reported the use of an oral glucose tolerance test to diagnose gestational diabetes due to the ignorance of healthcare providers. These findings suggest it remains important to share information regarding medication and MBS.

To report and share drug-related problems, most healthcare providers believe it will be best to report these to the pharmacovigilance center. This belief contrasts with our hypothesis of adverse events being underreported here. Many healthcare providers suggest reporting drug-related problems to the national registry for MBS patients. In the Netherlands, the nationwide Dutch Audit for Treatment of Obesity (DATO) has been established as a registry for patients with MBS [10]. Until now, drug-related problems cannot be registered here, while a national registry could be a convenient place to document changed drug effects. However, this registry is only accessible for bariatric surgeons only.

To improve the reporting process of changed drug effects after MBS, we propose healthcare professionals document these in systems such as a national registry or pharmacovigilance database. In this registry or pharmacovigilance database, additional information can be added such as the type of MBS and the year of surgery. One has to keep in mind that registrations in multiple systems need to be monitored from time to time and linked to each other to obtain valuable information that would have otherwise been missed. We suggest creating a simple format in both the national registry and the pharmacovigilance database to stimulate reporting by healthcare professionals. Furthermore, it is also important to document the observed changes in the patient’s electronic health record and transfer this information to other healthcare professionals of the patient.

As a strength, this survey was not limited to one field of healthcare professionals. It rather provides a broad overview of the beliefs, attitudes, and experiences of different professionals who are treating post-bariatric surgery patients. However, as in any other questionnaire with no incentive for participating in the survey, there is a risk for systematic bias as individuals with a strong opinion in both a negative and positive way are most likely to respond. Furthermore, more pharmacists responded to this survey. Moreover, the generalizability of our findings in this national cohort to other healthcare professionals treating post-bariatric surgery patients remains to be determined. Lastly, the low response rate is attributable to the privacy policy of the professional societies, as the survey could not be sent to all individuals directly.

This current survey identified that the majority of healthcare providers in different fields demand for additional training on pharmacotherapy in post-bariatric surgery patients. In contrast to previously identified knowledge gaps for MBS in general and bariatric surgeons [11, 12], we were not able to collect precise knowledge gaps of the respondents, since this survey did not ask specifically for input on training. However, there were no common patterns recognized in the input provided, indicating that there might be a general need for education on pharmacotherapy in post-bariatric surgery patients.

## Conclusions

This study shows that healthcare professionals in pharmacy, GP practice, internal medicine, and bariatric surgery believe that MBS is an important patient feature to consider when prescribing medicine. Not all healthcare professionals think they are competent to do so adequately. To improve this, information on changed drug effects after MBS should be more documented. New information on changed drug effects should be reported to resources and widely shared among healthcare professionals.

### Supplementary Information

Below is the link to the electronic supplementary material.

Supplementary file1 (DOCX 29.3 KB)

Supplementary file2 (DOCX 40.3 KB)

Supplementary file3 (DOCX 109 KB)

## Data Availability

The data that support the findings of this study are available from the corresponding author on reasonable request. The data are not publicly available due to privacy or ethical restrictions.
