# Supplementary material for: A Dutch Survey on Medication Adjustments after Metabolic and Bariatric Surgery: Experiences of Bariatric Surgeons, Internists, Pharmacists, and General Practitioners
Source: Obes Surg. 2024 Apr 2;34(5):1778–85. doi: 10.1007/s11695-024-07197-2 (PMC11031431; doi:10.1007/s11695-024-07197-2)
Supplement: Supplementary file 1 — Supplementary file1 (DOCX 29.3 KB) [file 11695_2024_7197_MOESM1_ESM.docx]

## **Supplementary Materials 1. English Translation of the Questionnaire**

**Introduction**

Dear healthcare professional:

Thank you for your time and effort to fill out this questionnaire. This questionnaire is intended for healthcare professionals who are working in the fields of general practitioners, internal medicine, bariatric surgery, and pharmacy.

The goal of this questionnaire is to characterize the state of the art in prescribing and monitoring drugs (in other words: pharmacovigilance).

In this questionnaire, these items will be pointed out:

- expectations and concerns about medication after bariatric surgery
- exchange of information
- monitoring of drugs
- education

It takes maximally 10 minutes to fill out this questionnaire.

Thank you for your participation.

**Questions**

A. Characteristics

| Q 1 | What is your sex? | Male |
| --- | --- | --- |
|  |  | Female |
|  |  | I prefer not to provide |
|  | | |
| Q 2 | What is your age? | 20-29 |
|  |  | 30-39 |
|  |  | 40-49 |
|  |  | 50-59 |
|  |  | ≥ 60 |
|  |  | I prefer not to provide |
|  | | |
| Q 3 | What field are you working in? | Surgery 🡪 go to Q 4 |
|  |  | General practitioner 🡪 go to Q 4 |
|  |  | Pharmacy |
|  |  | Internal medicine 🡪 go to Q 4 |
|  |  | Other, i.e.,… 🡪 go to Q 4 |
|  | | |
| Q 3a | In what setting are you working? | Hospital pharmacy |
|  |  | Community pharmacy |
|  |  | Outpatient pharmacy |
|  |  | Other, i.e.,… |
|  | | |
| Q 4 | What is your function? | Medical specialist |
|  |  | Resident or Junior |
|  |  | Nursing specialist |
|  |  | Nurse |
|  |  | Other, i.e.,… |
|  | | |
| Q 5 | How often do you provide care to a patient with post-bariatric surgery? | Daily or almost daily |
|  |  | Often |
|  |  | Sometimes |
|  |  | Never or rarely |

B. Expectations about Bariatric Surgery

| Q 6 | Do you expect a bariatric surgical procedure to influence the effect of medication? | Yes |
| --- | --- | --- |
|  |  | No |
|  |  | I do not know |
|  | | |
| Q 7 | To what extent do you think healthcare professionals should take into account the fact that a patient has undergone bariatric surgery when prescribing drugs? | Not at all |
|  |  | Sometimes |
|  |  | Often |
|  |  | Always |
|  | | |
| Q 8 | To what extent do you think a patient will receive better or safer pharmacotherapy when prior bariatric surgery status is taken into account? | Not at all |
|  |  | Sometimes |
|  |  | Often |
|  |  | Always |
|  | | |

C. Concerns about Medication after Bariatric Surgery

| Q 9 | To what extent do you worry that no suitable drugs are available for a patient who has undergone a bariatric surgical procedure? | Not at all |
| --- | --- | --- |
|  |  | Sometimes |
|  |  | Often |
|  |  | Always |
|  | | |
| Q 10 | To what extent do you worry about adjusted dosing in patients after bariatric surgery? | Not at all |
|  |  | Sometimes |
|  |  | Often |
|  |  | Always |
|  | | |
| Q 11 | To what extent do you think your patients worry about adjusted dosing after bariatric surgery? | Not at all |
|  |  | Sometimes |
|  |  | Often |
|  |  | Always |
|  | | |

D. Exchange of Information after Bariatric Surgery

| Q 12 | Have you registered ‘bariatric surgery’ as a contraindication in your prescribing system or pharmacy information system? | Yes |
| --- | --- | --- |
|  |  | No |
|  |  | I do not know |
|  | | |
| Q 13 | To what extent do you think it is important that the contraindication ‘bariatric surgery’ is known by other healthcare professionals of a patient? | Not important at all |
|  |  | Slightly important |
|  |  | Important |
|  |  | Very important |
|  | | |
| Q 14 | To what extent do you worry about privacy (General Data Protection Regulation) regarding sharing the contraindication bariatric surgery? | Not at all |
|  |  | Sometimes |
|  |  | Often |
|  |  | Always |
|  | | |

E. Monitoring of Pharmacotherapy after Bariatric Surgery / Pharmacovigilance

| Q 15 | In the last twelve months, have you prescribed or dispensed any medication to a patient with bariatric surgery, that had better been not prescribed? | Yes |
| --- | --- | --- |
|  |  | No |
|  |  | I do not know |
|  | | |
| Q 16 | Are you aware that medication advice after bariatric surgery is already implemented in the electronic prescribing systems and pharmacy information systems? | Yes |
|  |  | No 🡪 Q 18 |
|  | | |
| Q 17 | The medication advice that I do receive during prescribing or processing a receipt has helped me to… | Increase the efficacy of a drug |
|  |  | Reduce side effects |
|  |  | Counsel patients about possible changes in the effects of drugs |
|  |  | Increase medication adherence |
|  |  | No, the advice has not helped me at all. |
|  |  | Otherwise, i.e. |
|  | | |
| Q 18 | Do you monitor the effects of drugs in post-bariatric surgery patients? | No 🡪 Q 20 |
|  |  | Sometimes, on indication |
|  |  | Often |
|  |  | Always |
|  | | |
| Q 19 | What type of monitoring do you perform?  (multiple options possible) | Check laboratory values, including drug levels |
|  |  | Monitoring the effect of drugs |
|  |  | Monitoring adverse events of drugs |
|  |  | Otherwise, i.e. |
|  | | |
| Q 20 | Have your patients experienced less efficacy of their drugs after a bariatric surgery? | Yes, i.e.,… |
|  |  | No |
|  | | |
| Q 21 | Have your patients experienced side effects or medication-related complications of their drugs after a bariatric surgery? | Yes, more side effects, i.e.,… |
|  |  | Yes, more complications, i.e.,… |
|  |  | Yes, more adverse events and complications, i.e… |
|  |  | No |
|  | | |
| Q 22 | Have you performed any interventions regarding drugs of your post-bariatric surgery patients (multiple answers possible)? | Yes, converted into another drug |
|  |  | Yes, decreased the dose |
|  |  | Yes, increased the dose |
|  |  | Yes, the same drug was converted into another formulation (i.e. liquid or extended-release) |
|  |  | Provided additional counseling |
|  |  | Performed additional monitoring, i.e. laboratory controls or check of drug levels |
|  |  | No |
|  | | |
| Q 23 | Do you document the changes that patients experience regarding drugs after bariatric surgery? | Yes |
|  |  | No 🡪 Q 24 |
|  | | |
| Q 23a | Where do you document the changes that patients experience post-bariatric surgery regarding the effects of drugs?  (multiple options possible) | In the electronic health record of the patient |
|  |  | In a medical letter to another healthcare professional |
|  |  | As a registered adverse event/allergy in the electronic prescribing or pharmacy information system |
|  |  | National pharmacovigilance center |
|  |  | Manufacturer |
|  |  | Publication (i.e., case report) |
|  |  | Otherwise, i.e.… |
|  | | |
| Q 24 | Where do you think information about changed efficacy, adverse events, and complications related to medication after a bariatric surgery ought to be documented?  (multiple options possible) | National registry of bariatric surgery |
|  |  | National pharmacovigilance center |
|  |  | Manufacturer |
|  |  | Only in the electronic health record of the patient |
|  |  | Otherwise, i.e. |
|  | | |
| Q 25 | Do you provide specific drug counseling to post-bariatric surgery patients? | No |
|  |  | Sometimes |
|  |  | Often |
|  |  | Yes, always |
|  | | |

F. Education

| Q 26 | Do you feel competent to prescribe or to provide advice regarding medication in post-bariatric surgery patients? | Yes |
| --- | --- | --- |
|  |  | No |
| Q 26a | Could you specify this answer? | I.e. |
|  | | |
| Q 27 | Have you followed any training regarding prescribing drugs or advising pharmacotherapy to post-bariatric surgery patients? | Yes |
|  |  | No |
|  | | |
| Q 28 | Do you feel the need for additional training on bariatric surgery and drugs? | Yes |
|  |  | No |
| Q 28a | Please elaborate on your answer (optional) | I.e. |
|  | | |
| Q 29 | Would you like to share something with us that we have not asked? | Yes, i.e.… |
|  |  | No |
|  | | |

Thank you for your participation in this questionnaire. The results will be processed anonymously.
