# Supplementary material for: A Dutch Survey on Medication Adjustments after Metabolic and Bariatric Surgery: Experiences of Bariatric Surgeons, Internists, Pharmacists, and General Practitioners
Source: Obes Surg. 2024 Apr 2;34(5):1778–85. doi: 10.1007/s11695-024-07197-2 (PMC11031431; doi:10.1007/s11695-024-07197-2)
Supplement: Supplementary file 2 — Supplementary file2 (DOCX 40.3 KB) [file 11695_2024_7197_MOESM2_ESM.docx]

## **Supplementary Materials 2. Questionnaires with Answers Provided**

A. Characteristics

| **Number** | **Question** | **Answer** | **N** | **%** |
| --- | --- | --- | --- | --- |
| Q 1 | What is your sex? | Male | 69 | 31.1 |
|  |  | Female | 150 | 67.6 |
|  |  | I prefer not to provide | 3 | 1.4 |
|  | | |  |  |
| Q 2 | What is your age? | 20-29 | 33 | 14.9 |
|  |  | 30-39 | 81 | 36.5 |
|  |  | 40-49 | 54 | 24.3 |
|  |  | 50-59 | 39 | 17.6 |
|  |  | ≥ 60 | 12 | 5.4 |
|  |  | I prefer not to provide | 3 | 1.4 |
|  | | |  |  |
| Q 3 | What field are you working in? | Surgery | 43 | 19.4 |
|  |  | General practitioner | 13 | 5.9 |
|  |  | Pharmacy | 138 | 62.2 |
|  |  | Internal medicine | 22 | 9.9 |
|  |  | Other, i.e.,… | 6 | 2.7 |
|  | | |  |  |
| Q 3a | In what setting are you working? | Hospital pharmacy | 64 | 46.7 |
|  |  | Community pharmacy | 51 | 37.2 |
|  |  | Outpatient pharmacy | 19 | 13.9 |
|  |  | Other, i.e.… | 4 | 2.9 |
|  | | |  |  |
| Q 4 | What is your function? | Medical specialist | 166 | 74.8 |
|  |  | Resident or Junior | 40 | 18.0 |
|  |  | Nursing specialist | 3 | 1.4 |
|  |  | Nurse | 0 | 0.0 |
|  |  | Other:   - Student - Pharmacist - Technician | 2  3  4 | 0.9  1.4  1.8 |
|  | | |  |  |
| Q 5 | How often do you provide care to a patient with post-bariatric surgery? | (Almost) daily | 55 | 24.8 |
|  |  | Often | 52 | 23.4 |
|  |  | Sometimes | 88 | 39.6 |
|  |  | (Almost) never | 27 | 12.2 |

B. Expectations about Bariatric Surgery

| **Number** | **Question** | **Answer** | **N** | **%** |
| --- | --- | --- | --- | --- |
| Q 6 | Do you expect a bariatric surgical procedure to influence the effect of medication? | Yes | 217 | 97.7 |
|  |  | No | 3 | 1.4 |
|  |  | I do not know | 2 | 0.9 |
|  | | |  |  |
| Q 7 | To what extent do you think healthcare providers should take into account the fact that a patient has undergone bariatric surgery when prescribing drugs? | Not at all | 1 | 0.5 |
|  |  | Sometimes | 22 | 10.2 |
|  |  | Often | 48 | 22.3 |
|  |  | Always | 144 | 67.0 |
|  | | |  |  |
| Q 8 | To what extent do you think a patient will receive better or safer pharmacotherapy when prior bariatric surgery status is taken into account? | Not at all | 1 | 0.5 |
|  |  | Sometimes | 56 | 26.2 |
|  |  | Often | 86 | 40.2 |
|  |  | Always | 71 | 33.2 |

C. Concerns about Medication after Bariatric Surgery

| **Number** | **Question** | **Answer** | **N** | **%** |
| --- | --- | --- | --- | --- |
| Q 9 | To what extent do you worry that no suitable drugs will be available for a patient who has undergone a bariatric surgical procedure? | Not at all | 95 | 45.0 |
|  |  | Sometimes | 114 | 54.0 |
|  |  | Often | 2 | 0.9 |
|  |  | Always | 0 | 0.0 |
|  | | |  |  |
| Q 10 | To what extent do you worry about adjusted dosing in patients after bariatric surgery? | Not at all | 33 | 15.6 |
|  |  | Sometimes | 136 | 64.5 |
|  |  | Often | 37 | 17.5 |
|  |  | Always | 5 | 2.4 |
|  | | |  |  |
| Q 11 | To what extent do you think your patients worry about adjusted dosing after bariatric surgery? | Not at all | 33 | 15.8 |
|  |  | Sometimes | 116 | 55.5 |
|  |  | Often | 57 | 27.3 |
|  |  | Always | 3 | 1.4 |

D. Exchange of Information after Bariatric Surgery

| **Number** | **Question** | **Answer** | **N** | **%** |
| --- | --- | --- | --- | --- |
| Q 12 | Have you registered ‘bariatric surgery’ as a contraindication in your prescribing system or pharmacy information system? | Yes | 132 | 63.5 |
|  |  | No | 66 | 31.7 |
|  |  | I do not know | 10 | 4.8 |
|  | | |  |  |
| Q 13 | To what extent do you think it is important that the contraindication ‘bariatric surgery’ is known by other healthcare providers of a patient? | Not important at all | 2 | 1.0 |
|  |  | Slightly important | 8 | 3.8 |
|  |  | Important | 60 | 28.8 |
|  |  | Very important | 138 | 66.3 |
|  | | |  |  |
| Q 14 | To what extent do you worry about privacy (General Data Protection Regulation) regarding sharing the contraindication bariatric surgery? | Not at all | 96 | 46.4 |
|  |  | Sometimes | 79 | 38.2 |
|  |  | Often | 26 | 12.6 |
|  |  | Always | 6 | 2.9 |

E. Monitoring of Pharmacotherapy after Bariatric Surgery / Pharmacovigilance

| **Number** | **Question** | **Answer** | **N** | | **%** |
| --- | --- | --- | --- | --- | --- |
| Q 15 | In the last twelve months, have you prescribed or dispensed any medication to a patient with bariatric surgery, that had better been not prescribed? | Yes | 36 | | 17.6 |
|  |  | No | 86 | | 42.2 |
|  |  | I do not know | 82 | | 40.2 |
|  | | |  | |  |
| Q 16 | Are you aware that medication advice after bariatric surgery is already implemented in the electronic prescribing systems and pharmacy information systems? | Yes | 149 | | 73.8 |
|  |  | No | 53 | | 26.2 |
|  | | |  | |  |
| Q 17 | The medication advice that I do receive during prescribing or processing a receipt has helped me to… | Increase the efficacy of a drug | 89 | | 44.1 |
|  |  | Reduce side effects | 75 | | 37.1 |
|  |  | Counsel patients about possible changes in the effects of drugs | 67 | | 33.2 |
|  |  | Increase medication adherence | 10 | | 5.0 |
|  |  | No, the advice has not  helped me at all. | 5 | | 2.5 |
|  |  | Otherwise:   - Switching of drugs - Answering questions from other healthcare professionals - Advice is not concrete - Making a more evidence-based decision - Not applicable / no experience | 1  3  2  1  11 | | 0.5  1.5  1.0  0.5  5.4 |
|  | | |  | |  |
| Q 18 | Do you monitor the effects of drugs in post-bariatric surgery patients? | No | 51 | | 25.9 |
|  |  | Sometimes, on indication | 110 | | 55.8 |
|  |  | Often | 21 | | 10.7 |
|  |  | Always | 15 | | 7.6 |
|  | | |  | |  |
| Q 19 | What type of monitoring do you perform?  (multiple options possible) | Check laboratory values, including drug levels | 98 | | 67.1 |
|  |  | Monitoring the effect of drugs | 103 | | 70.5 |
|  |  | Monitoring adverse events of drugs | 67 | | 45.9 |
|  |  | Otherwise:   - Switching drugs / contraindication of drugs - Check of dosage | 2  1 | | 1.4  0.7 |
|  | | |  | |  |
| Q 20 | Have your patients experienced less efficacy of their drugs after a bariatric surgery? | Yes:  - psychotropic drugs  - oral contraceptive drugs  - antiviral/antimicrobial drugs  - anticoagulants  - metabolic drugs  - vitamins/minerals  - various drugs without specification  - other | 41  15  3  2  2  8  7  8  2 | | 21.1 |
|  |  | No | 153 | | 78.9 |
|  | | |  | |  |
| Q 21 | Have your patients experienced side effects or medication-related complications of their drugs after a bariatric surgery? | Yes, more side effects, i.e.,.… | 16 | | 8.3 |
|  |  | Yes, more complications, i.e.,… | 14 | | 7.3 |
|  |  | Yes, more adverse events and complications, i.e.,… | 10 | | 5.2 |
|  |  | No | 153 | | 79.3 |
|  | | |  | |  |
| Q 22 | Have you performed any interventions regarding drugs of your post-bariatric surgery patients (multiple answers possible)? | Yes, converted into another drug | 115 | | 59.6 |
|  |  | Yes, decreased the dose | 37 | | 19.2 |
|  |  | Yes, increased the dose | 47 | | 24.4 |
|  |  | Yes, the same drug was converted into another formulation (i.e., liquid or extended-release) | 72 | | 37.3 |
|  |  | Provided additional counseling | 67 | | 34.7 |
|  |  | Performed additional monitoring, i.e., laboratory controls or check of drug levels | 59 | | 30.6 |
|  |  | No | | 31 | 16.1 |
|  | | |  | |  |
| Q 23 | Do you document the changes that patients experience regarding drugs after bariatric surgery? | Yes | | 91 | 47.9 |
|  |  | No | | 99 | 52.1 |
|  | | |  | |  |
| Q 23a | Where do you document the changes that patients experience post-bariatric surgery regarding the effects of drugs?  (multiple options possible) | In the electronic health record of the patient | | 87 | 95.6 |
|  |  | In a medical letter to another healthcare professional | | 20 | 22.0 |
|  |  | As a registered adverse event/allergy in the electronic prescribing or pharmacy information system | | 8 | 8.8 |
|  |  | National pharmacovigilance center | | 3 | 3.3 |
|  |  | Manufacturer | | 2 | 2.2 |
|  |  | Publication (i.e., case report) | | 4 | 4.4 |
|  |  | Otherwise, i.e.… | | 3 | 1.4 |
|  | | | |  |  |
| Q 24 | Where do you think information about changed efficacy, adverse events, and complications related to medication after a bariatric surgery ought to be documented?  (multiple options possible) | National registry of bariatric surgery | | 73 | 38.4 |
|  |  | National pharmacovigilance center | | 98 | 51.6 |
|  |  | Manufacturer | | 49 | 25.8 |
|  |  | Only in the electronic health record of the patient | | 68 | 35.8 |
|  |  | Otherwise:   - Drug database - And in the electronic health record of the patient - Pharmacy / pharmacist - Literature or reference work - Other | | 22  5  2  7  3 | 11.6  2.6  1.1  3.7  1.6 |
|  | | | |  |  |
| Q 25 | Do you provide specific drug counseling to post-bariatric surgery patients? | No | | 48 | 25.4 |
|  |  | Sometimes | | 86 | 45.5 |
|  |  | Often | | 33 | 17.5 |
|  |  | Yes, always | | 22 | 11.6 |

F. Education

| **Number** | **Question** | **Answer** | **N** | **%** |
| --- | --- | --- | --- | --- |
| Q 26 | Do you feel competent to prescribe or to provide advice regarding medication in post-bariatric surgery patients? | Yes | 125 | 66.5 |
|  |  | No | 63 | 33.5 |
|  | | |  |  |
| Q 27 | Have you followed any training regarding prescribing drugs or advising pharmacotherapy to post-bariatric surgery patients? | Yes | 63 | 33.9 |
|  |  | No | 123 | 66.1 |
|  | | |  |  |
| Q 28 | Do you feel the need for additional training on bariatric surgery and drugs? | Yes | 122 | 65.6 |
|  |  | No | 64 | 34.4 |
