# Supplementary material for: A Dutch Survey on Medication Adjustments after Metabolic and Bariatric Surgery: Experiences of Bariatric Surgeons, Internists, Pharmacists, and General Practitioners
Source: Obes Surg. 2024 Apr 2;34(5):1778–85. doi: 10.1007/s11695-024-07197-2 (PMC11031431; doi:10.1007/s11695-024-07197-2)
Supplement: Supplementary file 3 — Supplementary file3 (DOCX 109 KB) [file 11695_2024_7197_MOESM3_ESM.docx]

**Supplementary Materials 3**


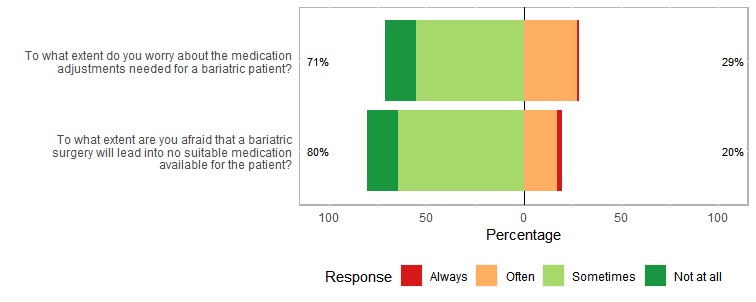


Figure S1. Likert plot of the concerns of healthcare providers related to MBS (N=197). The size of the bar is proportional to the number of respondents


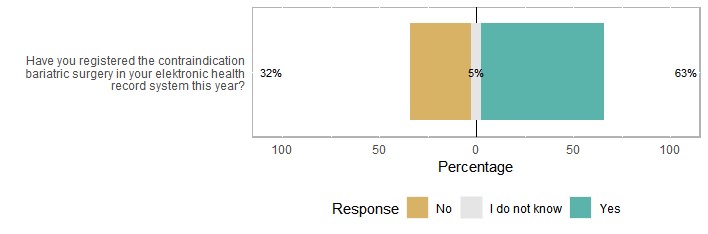


Figure S2. Likert plot of registering the contraindication bariatric surgery in the last year (N=204). The size of the bar is proportional to the number of respondents


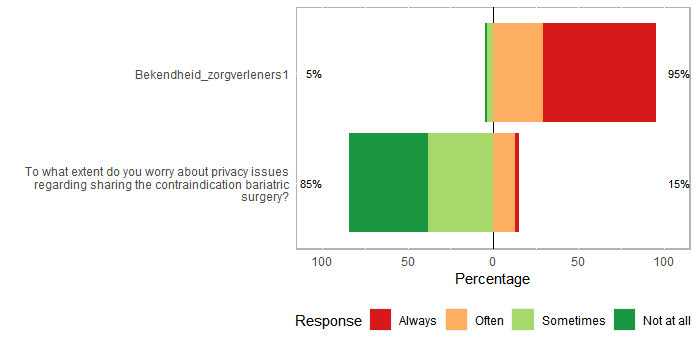


Figure S3. Likert plot of concerns about privacy issues regarding sharing the contraindication bariatric surgery (N=208). The size of the bar is proportional to the number of respondents


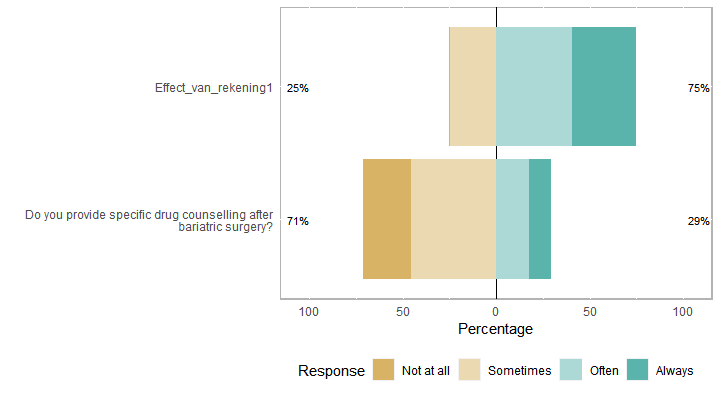


Figure S4. Likert plot about specific drug counseling after MBS (N=197). The size of the bar is proportional to the number of respondents


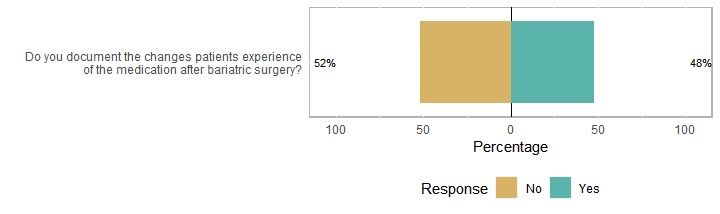


Figure S5. Likert plot about documentation of patients’ changed experience of medication after MBS (N=190). The size of the bar is proportional to the number of respondents
